# A model worker: Multifaceted modulation of AUXIN RESPONSE FACTOR3 orchestrates plant reproductive phases

**DOI:** 10.3389/fpls.2023.1123059

**Published:** 2023-02-27

**Authors:** Yunze Fu, Hao Zhang, Yuru Ma, Cundong Li, Ke Zhang, Xigang Liu

**Affiliations:** ^1^ State Key Laboratory of North China Crop Improvement and Regulation, Key Laboratory of Crop Growth Regulation of Hebei Province, College of Agronomy, Hebei Agricultural University, Baoding, Hebei, China; ^2^ Ministry of Education Key Laboratory of Molecular and Cellular Biology, Hebei Key Laboratory of Molecular and Cellular Biology, College of Life Sciences, Hebei Normal University, Hebei Collaboration Innovation Center for Cell Signaling, Shijiazhuang, China; ^3^ State Key Laboratory of North China Crop Improvement and Regulation, Key Laboratory of Hebei Province for Plant Physiology and Molecular Pathology, College of Life Sciences, Hebei Agricultural University, Baoding, China

**Keywords:** *Arabidopsis thaliana*, auxin, AUXIN RESPONSE FACTOR3, reproductive phase, meristem homeostasis, patterning formation

## Abstract

The key phytohormone auxin is involved in practically every aspect of plant growth and development. Auxin regulates these processes by controlling gene expression through functionally distinct AUXIN RESPONSE FACTORs (ARFs). As a noncanonical ARF, ARF3/ETTIN (ETT) mediates auxin responses to orchestrate multiple developmental processes during the reproductive phase. The *arf3* mutation has pleiotropic effects on reproductive development, causing abnormalities in meristem homeostasis, floral determinacy, phyllotaxy, floral organ patterning, gynoecium morphogenesis, ovule development, and self-incompatibility. The importance of ARF3 is also reflected in its precise regulation at the transcriptional, posttranscriptional, translational, and epigenetic levels. Recent studies have shown that ARF3 controls dynamic shoot apical meristem (SAM) maintenance in a non-cell autonomous manner. Here, we summarize the hierarchical regulatory mechanisms by which ARF3 is regulated and the diverse roles of ARF3 regulating developmental processes during the reproductive phase.

## 1 Introduction

​Classically, auxin regulates gene expression by controlling the activity of AUXIN RESPONSE FACTORs (ARFs) through the Aux/IAA pathway. The Arabidopsis (*Arabidopsis thaliana*) genome encodes 23 ARFs that can be divided into three subclasses: A, B, and C (Guilfoyle and Hagen, 2007; Finet et al., 2013; Roosjen et al., 2018). ARF3/ETTIN (ETT) belongs to class B and is an important regulator of many developmental processes. Located on chromosome 2, *ARF3* contains 10 exons and 9 introns, has a total length of 3170 bp, and encodes a protein that is 608 amino acids long. Most ARFs possess three major domains: an N-terminal DNA-binding domain (DBD), with higher affinity for the DNA-binding motifs TGTCGG and TGTCTC; a middle region (MR), which can be used as a reference for determining activators and repressors in the ARF family; and a C-terminal Phox and Bem1 domain (PB1 or domain III/IV). The PB1 domain allows ARFs to form homodimers and heterodimers with other ARFs (Boer et al., 2014; Weijers and Wagner, 2016; Roosjen et al., 2018). Canonically, ARF activity is inhibited by Aux/IAA proteins when auxin levels are low, but this repression is released when auxin concentrations increase (Vernoux et al., 2011; Calderon Villalobos et al., 2012). ARF3, ARF13, ARF17, and ARF23 are distinct in that they lack the PB1 domain (Guilfoyle and Hagen, 2007; Guilfoyle, 2015; Li et al., 2016b), raising the possibility that auxin may not regulate ARF3 activity through the ARF-Aux/IAA pathway (Chandler, 2016). However, a unique C-terminal ETT-specific domain (ES domain) in ARF3 can sense auxin signals (Simonini et al., 2016), through binding auxin directly, and that this interaction determines the expression of ARF3 target genes (Kuhn et al., 2020).

A flowering plant goes through four stages in its life: embryonic development, vegetative growth, reproductive growth, and senescence. The reproductive growth phase is a critical period for plant fitness and is a focal point for biologists and breeders alike (Roeder and Yanofsky, 2006; Alvarez-Buylla et al., 2010). In Arabidopsis, reproduction begins with the transition to flowering, in which the shoot apical meristem (SAM) is transformed into an inflorescence meristem (IM) that continuously forms an orderly arrangement of flower primordia around a central axis (Traas, 2013; Li and He, 2020). IM (or SAM) homeostasis is the basis of indeterminate growth in plants and is modulated by phytohormones, specific genes, and the environment (Lee et al., 2019; Ma et al., 2019). Floral primordia differentiate into a specific number of floral organs at specific locations (Sessions et al., 1997; Thomson and Wellmer, 2019). Floral meristem (FM) determinacy occurs after the initiation of carpel primordia, which is required for normal gynoecium development (Cao et al., 2015; Sun and Ito, 2015). Many mutants with FM determinacy defects exhibit abnormal gynoecia and thus abnormal ovule development (Liu et al., 2011; Liu et al., 2014; Li et al., 2016a). The formation of male and female gametes as well as successful fertilization are crucial for the development of fruit (Drews and Koltunow, 2011; Verma, 2019). Genetic and phylogenetic analyses have confirmed that there is widespread functional redundancy among ARF family members; most *arf* single mutants display no obvious phenotypes (Remington et al., 2004; Tian et al., 2004; Okushima et al., 2005). However, *arf3* mutations have pleiotropic effects on reproductive development, causing abnormalities in meristem homeostasis, floral determinacy, patterning formation, gynoecium morphogenesis, ovule development, and self-incompatibility (Sessions et al., 1997; Tantikanjana and Nasrallah, 2012; Liu et al., 2014; Chandler, 2016; Su et al., 2017). These observations suggest that ARF3 has a special role in auxin signaling and developmental responses.

In a regulatory network, the critical aspects tend to be more precisely and intricately regulated. In addition to mediating auxin signaling using a noncanonical auxin-sensing mechanism, *ARF3* transcription is regulated by changes in DNA methylation as well as changes in the abundance of many transcription factors, such as ASYMMETRIC LEAVES1 (AS1)-AS2, APETALA2 (AP2), and AGAMOUS (AG). *ARF3* transcript abundance is further fine-tuned by microRNAs (miRNAs) and trans-acting short-interfering RNA–auxin response factors (tasiR-ARFs) at the posttranscriptional level (Marin et al., 2010; Liu et al., 2014; Machida et al., 2015; Simonini et al., 2016). ARF3 translation is controlled by upstream open reading frames (uORFs)-mediated translation reinitiation, followed by intercellular migration that leads to a precise distribution of the protein (Nishimura et al., 2005; Liu et al., 2014). Furthermore, ARF3 has a number of interacting factors that allow it to execute complex and diverse biological functions (Bao et al., 2010; Kelley et al., 2012).

## 2 Regulation of ARF3 at multiple levels

*ARF3* expression and activity of the encoded protein are controlled at multiple levels, including transcriptional, posttranscriptional, translational, and epigenetic levels. Furthermore, ARF3 regulates plant development in both cell- and non-cell-autonomous manners. In this section, the modulation and mechanisms of ARF3 at various levels are summarized.

### 2.1 Regulation of *ARF3* transcription

**Figure 1:**
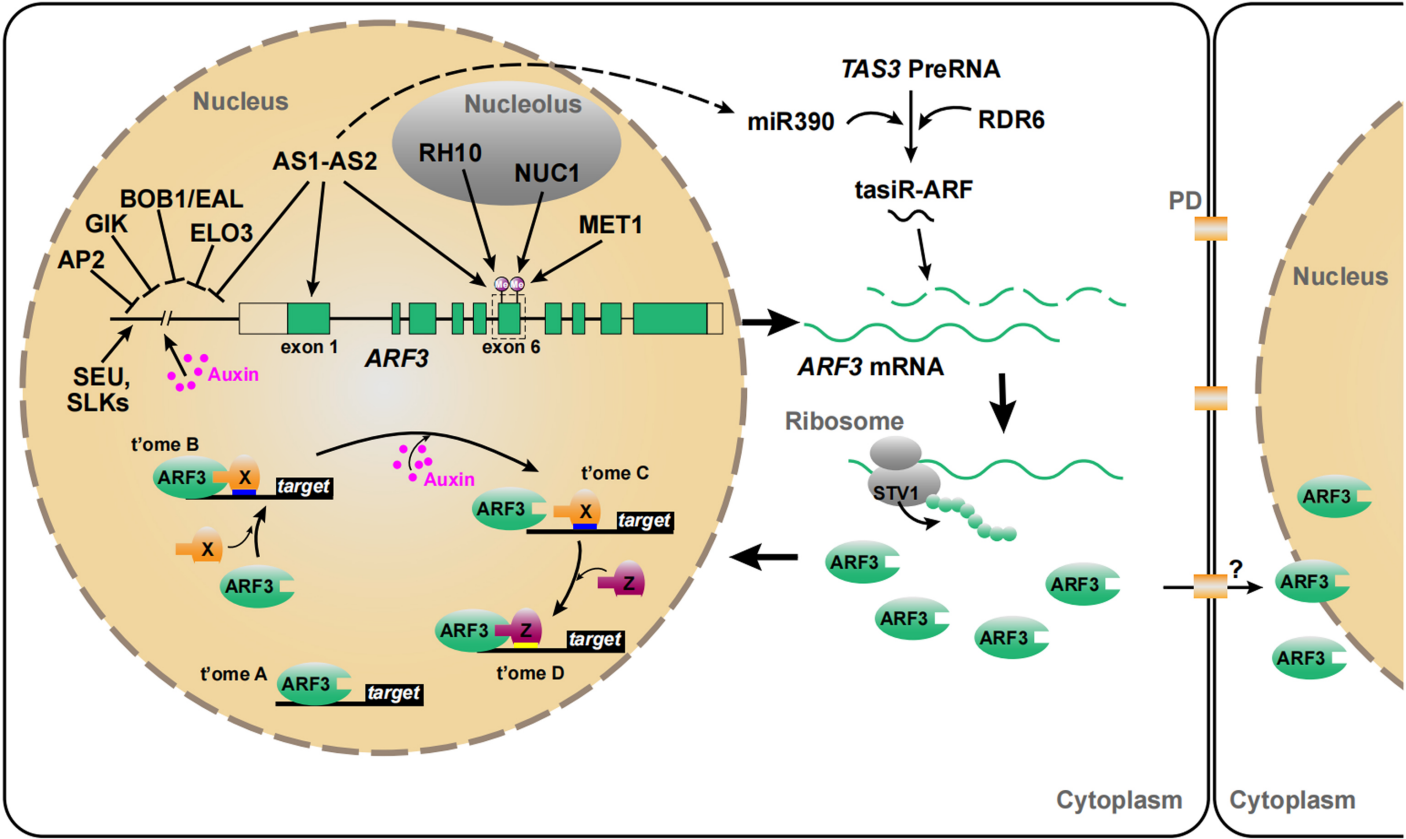
Regulation of ARF3 at multiple levels. ARF3 is affected by transcriptional regulation, tasiR-ARF, translation reinitiation, and DNA methylation (Nishimura et al., 2005; Vial-Pradel et al., 2018; Iwakawa et al., 2020). ARF3 regulates its target genes in two ways, either dependent on auxin or independent of auxin (Simonini et al., 2017). ARF3 can also migrate intercellularly (Zhang et al., 2022). The *ARF3* gene, mRNA, and protein are in green. Auxin is in magenta. t’ome A/B/C/D, transcriptome A/B/C/D. “X” and “Z”, the partners of ARF3. PD, plasmodesmata (Whether ARF3 travels from cell to cell *via* PD remains to be confirmed).

At the transcriptional level, in addition to being induced by auxin, *ARF3* is directly repressed by the AS1–AS2 complex, which is involved in the establishment of leaf polarity (Iwakawa et al., 2020). GIANT KILLER (GIK), containing an AT-hook DNA binding motif, is a target of AG that can bind to the *ARF3* promoter and inhibit its transcription, indicating that GIK mediates the transcriptional activation of *ARF3* depending on AG activity (Ng et al., 2009; Zhang et al., 2018b). The AP2 domain-containing protein APETALA2 (AP2) also directly represses *ARF3* transcription to mediate its own role in floral determinacy (Liu et al., 2014; **Figure 1**; details below).

### 2.2 Regulation of ARF3 by tasiR-ARF

ARF3 is also regulated at the post-transcriptional level by endogenous tasiR-ARF, encoded by the trans-acting siRNA3 (*TAS3*) locus, which shares a 21- and 22-nt region of sequence similarity with *ARF3* and *ARF4*, respectively (Williams et al., 2005; Hunter et al., 2006b; **Figure 1**). The biogenesis of tasiR-ARF shares similarities with both the siRNA and miRNA pathways. tasiR-ARF production begins with specific microRNA 390 (miR390)-mediated cap and polyadenosine cleavage of the primary *TAS3* transcripts (pri-TAS3) (Vaucheret, 2005; Allen and Howell, 2010; Yoshikawa, 2013). Subsequently, the 5′ cleavage fragments are transformed into double-stranded RNAs (dsRNAs) by RNA-DEPENDENT RNA POLYMERASE 6 (RDR6) and processed by DICER-LIKE 4 to form tasiRNA3 (Vaucheret, 2005; Yoshikawa, 2013). Trans-acting siRNA-mediated repression of *ARF3* is involved in the regulation of juvenile development (Peragine et al., 2004), heteroblasty (Hunter et al., 2006), morphology and patterning of leaves and floral organs (Fahlgren et al., 2006; Liu et al., 2014), ovule development (Su et al., 2017), self-incompatibility (Tantikanjana and Nasrallah, 2012), and lateral root growth (Marin et al., 2010).

### 2.3 Regulation of ARF3 by translation reinitiation

uORFs have a negative effect on gene translation, but this can be mitigated by ribosomal translation reinitiation (von Arnim et al., 2014). Translation reinitiation is a regulatory mechanism that controls the expression of specific genes involved in developmental programs and responses to environmental signals (Nishimura et al., 2005; Rahmani et al., 2009; Ivanov et al., 2010). *ARF3* is predicted to contain two uORFs in the 5′ untranslated region (Nishimura et al., 2005). *SHORT VALVE1* (*STV1*), encoding the large ribosomal subunit protein Ribosomal protein L24B (RPL24B), affects ARF3 translation by mediating translation reinitiation and is associated with apical–basal patterning in gynoecia (Nishimura et al., 2005). *STV1* transcripts are distributed throughout shoot apices and in young developing flowers, but are most abundant in vigorously dividing cells such as IMs, flower meristems (FMs), and flower organ primordia. The gynoecia of *stv1* mutants display a similar phenotype to *ett-2* mutants, which contain a weak allele of *ett*. *stv1-1 ett-2* double mutants display even more serious defects in their gynoecia, including fewer ovaries and morphological abnormalities (Nishimura et al., 2005). In addition, ARF3 uORFs are partially responsible for auxin-related defects of root development in *rpl4d* and *rpl5a* (Rosado et al., 2012; **Figure 1**). Auxin might efficiently use ribosomes to fulfill global and specialized regulation during plant development. (Rosado et al., 2012; **Figure 1**)

### 2.4 Regulation of *ARF3* by DNA methylation

DNA methylation is an epigenetic modification involved in gene regulation, genome stability, and plant development (Bartels et al., 2018; Zhang et al., 2018a). The cytosine residues at CG sites in exons 6 and 10 of *ARF3* are often strongly methylated in wild-type plants, suggesting that *ARF3* transcription is regulated by epigenetic modification (Zhang et al., 2006; Cokus et al., 2008). AS1–AS2, a nuclear protein complex, plays an important role in maintaining DNA methylation within the *ARF3* coding region to regulate adaxial–abaxial partitioning in leaves (Takahashi et al., 2013; Machida et al., 2015; Iwakawa et al., 2020). Genetic analysis revealed that *ARF3* and *ARF4* are regulated by the modifiers BOBBER1 (BOB1) and ELONGATA3 (ELO3) along with AS1–AS2 (Takahashi et al., 2013). In *as1* and *as2* mutants, the cytosine residues in CG pairs have a lower methylation level in exon 6 of *ARF3*, and ARF3 transcript levels increased correspondingly. An *AS2-eoe/as2-1*, ectopic overexpression line, however, had similar levels of CG methylation as the wild type (Iwasaki et al., 2013). *NUCLEOLIN1* (*NUC1*) and *RNA HELICASE10* (*RH10*), encoding nucleolus-localized proteins, are also involved in the epigenetic repression of *ARF3 via* gene body DNA methylation through several independent pathways (Vial-Pradel et al., 2018). *METHYL TRANSFERASE1* (*MET1*), encoding a cytosine methyltransferase, can also repress *ARF3* transcription in shoot apices by maintaining the CG methylation status of *ARF3* (Ronemus et al., 1996; Iwasaki et al., 2013; **Figure 1**).

### 2.5 ARF3 functions as a non-cell-autonomous transcription factor

Non-cell-autonomous transcription factors can function as cell-to-cell communication signals and play pivotal roles in most processes related to the formation and development of plant organs (Han et al., 2014). ARF3 protein has a distribution pattern distinct from that of its transcript in the meristem, indicating that it is capable of intercellular migration (Liu et al., 2014). This notion has been verified by comparing the distribution patterns of *ARF3:ARF3–GFP* and *ARF3:ARF3-nls-GFP* (in which GFP contains a nuclear localization tag, or nls) in IMs. Longitudinal sections imaged with confocal microscopy exhibit ARF3-GFP signals throughout the IM, but ARF3-nls-GFP signals could only be detected in the peripheral zone (PZ) and central zone (CZ) of meristems (Zhang et al., 2022), suggesting that ARF3 functions in a non-cell-autonomous manner (see section 2 for details).

## 3 ARF3 mediates auxin responses

ARF3 does not mediate auxin signaling through the canonical ARF–Aux/IAA pathway since it lacks the PB1 domain (Guilfoyle and Hagen, 2007; Vernoux et al., 2011; Calderon Villalobos et al., 2012; Chandler, 2016). Nonetheless, exogenous auxin was shown to up-regulate *ARF3* expression during floral development and *de novo* organ regeneration processes (Cheng et al., 2013; Zhang et al., 2018b). *ARF3* mRNA has been detected in the meristem periphery and in organ primordia, which corresponds with auxin maxima and suggests that *ARF3* expression depends on auxin activity (Sessions et al., 1997; Vernoux et al., 2011).

Recently, a noncanonical auxin-sensing mechanism was proposed to explain how the auxin-dependent modulation of ARF3 activity regulates target gene expression (Simonini et al., 2016; Simonini et al., 2017; Simonini et al., 2018; Kuhn et al., 2020). The ETT-ES domain directly binds to auxin to perceive auxin and affects the expression of ARF3 target genes. (Kuhn et al., 2020). Simonini et al. found that ARF3 physically interacts with INDEHISCENT (IND) in an auxin-sensitive manner to control polarity at the gynoecium apex (Simonini et al., 2016). Furthermore, Kuhn et al., showed under low auxin conditions an ARF3- TOPLESS (TPL)- HISTONE DEACETYLASE 19 (HDA19) complex binds to the promoter of *PINOID* (*PID*) and *HECATE1* (*HEC1*) keeping their chromatin environments repressed, through de-acetylation, while high nuclear auxin concentrations abolish the ARF3-TPL-HDA19 complex through direct ARF3-auxin interaction (Kuhn et al., 2020). Simonini et al. proposed that auxin affects ARF3 action by controlling its dimerization and transcription. For example, ARF3 regulates target gene expression alone (transcriptome A, t’ome A), and ARF3 function by coupling with process-specific protein partners (“x”) (t’ome B); the dimerization of ARF3 with partners is reversed as auxin concentrations increase, leading to a different transcriptional outcome (t’ome C); and the released ARF3 may interact with new partners (“Z”) (t’ome D) (Simonini et al., 2017; **Figure 1**).

## 4 ARF3 influences meristem fate

Plant growth and development are based on the precise control of meristem fate. For example, the SAM needs to maintain homeostasis to continuously produce lateral organs, while FM activity requires appropriate termination, known as FM determinacy, to form the correct number of whorls and floral organs (Lee et al., 2019; Chang et al., 2020). Many regulatory factors, including plant hormones, transcription factors, secreted peptides, and environmental signals, work together to construct a complex regulatory network that controls meristem fate (Cao et al., 2015; Sun and Ito, 2015). Recent studies revealed that ARF3-mediated auxin signaling contributes to the fine-tuning of meristem homeostasis and FM determinacy (Liu et al., 2014; Zhang et al., 2018b; Zhang et al., 2022).

### 4.1 Shoot apical meristem homeostasis

Meristems possess the ability to self-renew and continuously produce new tissues and organs (Zhang and Yu, 2014). To maintain meristem homeostasis, dynamic signals and gene expression need to be precisely regulated both spatially and temporally (Lee et al., 2019). Auxin plays a critical role in specifying the fate of organ primordia in the SAM (Vernoux et al., 2011; Janocha and Lohmann, 2018). High levels of auxin in the peripheral regions of the SAM mediate primordial differentiation, but low levels are required in the organizing center (OC) and CZ (Schaller et al., 2015; Shi et al., 2018; Ma et al., 2019). WUSCHEL (WUS), a homeodomain transcription factor, mediates stem cells’ resistance to auxin but allows low levels to persist in the CZ to ensure stem cell maintenance (Ma et al., 2019). Auxin also interacts synergistically with cytokinin (CK) signals to regulate WUS expression in the SAM and FMs (Schaller et al., 2015; Zhang et al., 2018b).

**Figure 2:**
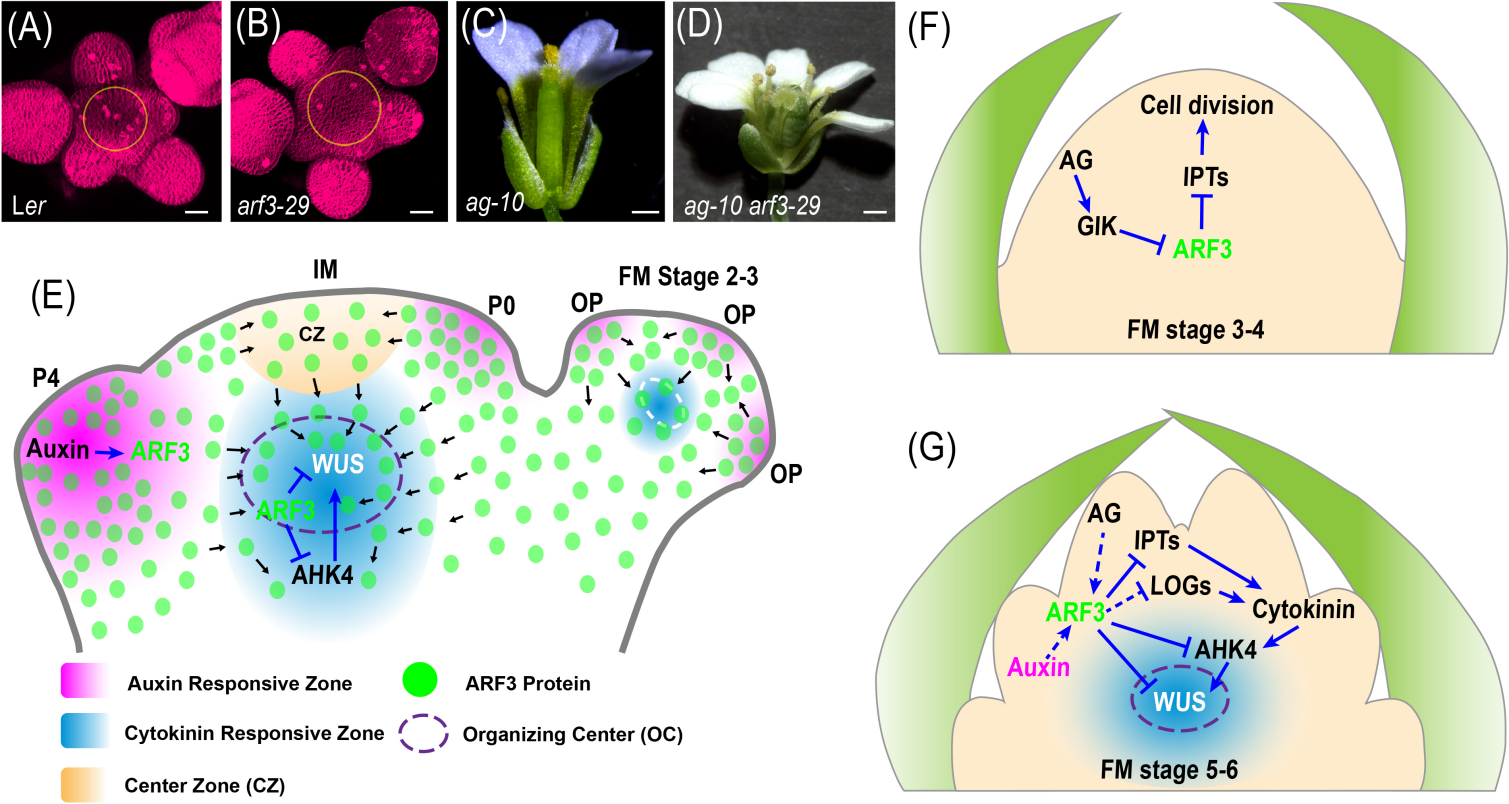
Models of SAM and FM activity control by ARF3. **(A, B)**, Representative images of SAM for the indicated genotypes (from Zhang et al., 2022, provided by Dr. Ke Zhang). **(C, D)**, Representative images of flower for the indicated genotypes. **(E)**, In the IM, auxin promotes ARF3, which migrates from the peripheral zone (PZ) to the organizing center (OC) where it directly represses AHK4 and WUS expression to control meristem activity in a non-cell-autonomous manner. Figures adapted from (Zhang et al., 2022). P0 and P4, primordia of flower buds at different developmental stages; OP, flower organ primordia. **(F)**, At stages 3-4 of floral development, AG indirectly represses *ARF3* expression through GIK, and consequently enhancing *IPTs* to promote cell division and maintain the meristem cell population, thereby favoring floral meristem (FM) maintenance. Figures adapted from (Zhang et al., 2018b) **(G)**, At stages 5-6 of floral development, auxin and AG promote *ARF3* expression, which in turn directly represses *IPTs*, and *AHK4* and indirectly represses *LOG* genes expression. The resulting inhibition of cytokinin activity is required for proper temporal termination of *WUS* expression during FM determinacy. Figures adapted from (Zhang et al., 2018b). Blue solid and dashed arrows indicate direct and indirect positive regulation, respectively. Blue solid and dashed block lines indicate direct and indirect negative regulation, respectively. Black arrows indicate the direction of protein movement.

However, how auxin, which is concentrated in the PZ, regulates cytokinin signaling and WUS expression in the OC remains additional research. Recent observations that ARF3 migrates between cells suggest that ARF3 may act as a messenger in the auxin-mediated regulation of WUS and cytokinin signaling (Lanctot, 2022; Zhang et al., 2022). *ARF3* transcript patterns coincide with auxin maxima in regions of primordia initiation, but ARF3 protein has been detected throughout the meristem (Liu et al., 2014; Simonini et al., 2017). Additionally, the transgenic expression of *ARF3:ARF3-nls-GFP* effectively prevents ARF3-nls-GFP from translocating into the OC from adjacent cells in the SAM (Zhang et al., 2022). Statistical analysis demonstrated that *arf3-29* mutants have larger SAMs than the wild type (**Figures 2A, B**), and *ARF3:ARF3-nls-GFP* can only partially rescue this mutant phenotype, suggesting that ARF3 protein migration is required for SAM activity. Furthermore, ARF3 intercellular migration is necessary for its inhibition of cytokinin signaling and *WUS* expression. Compared with *ARF3:ARF3-GFP arf3-29*, ARF3-nls-GFP enrichment at the *ARABIDOPSIS HISTIDINE KINASE4* (*AHK4*) and *WUS* loci was significantly reduced, while the transcript levels of *AHK4* and *WUS-DsRed* increased. These findings reveal a network of ARF3-mediated crosstalk between phytohormones and gene expression that maintains meristem homeostasis (Lanctot, 2022; Zhang et al., 2022; **Figure 2E**).

### 4.2 Floral meristem determinacy

Unlike stem cells in the SAM, floral stem cells cease to be stem cells after generating carpel primordia (Liu et al., 2011). At stages 3–4 of floral development, AG indirectly represses *ARF3* expression through GIK (Ng et al., 2009) and other factors, consequently enhancing *ISOPENTENYLTRANSFERASE* (*IPT*) and cell cycle gene expression to promote cell division and maintain the meristem cell population, thereby favoring FM maintenance (**Figure 2F**) (Zhang et al., 2018b). However, some mutations uncouple the formation of carpel primordia from the loss of stem cell identity, suggesting that the termination of floral stem cell maintenance is not synonymous with the differentiation of stem cells into carpel cells (Prunet et al., 2008; Sun et al., 2009; Ji et al., 2011). The orchestration of multiple regulators ensures precise timing of stem cell termination (Sun and Ito, 2010; Xu et al., 2019). In spite of FM determinacy being normal in *arf3-29* mutants, this mutation can enhance FM determinacy defects in *ag-10* plants, which harbor a weak *ag* allele (Liu et al., 2014). *arf3-29 ag-10* double mutants have bulged and unfused carpels with hyperplasia tissue on the inside, although most of the siliques in *ag-10* single mutant plants are morphologically similar to wild-type siliques (**Figures 2C, D**). *WUS* is a key regulator of stem cell maintenance in both the SAM and FM (Laux et al., 1996; Cao et al., 2015). *WUS* expression was undetectable at stage 6 of floral development in wild-type flowers and 90% of *ag-10* flowers have normal *WUS* expression patterns (Yumul et al., 2013; Liu et al., 2014). All *ag-10 arf3-29* double mutant flowers, however, had prolonged *WUS* expression until or beyond stage 7 (Liu et al., 2014). ARF3 integrates the functions of AP2 and AG to repress *WUS* expression in floral meristem determinacy (Liu et al., 2014; Shang et al., 2019; Thomson and Wellmer, 2019). AG acts independently of AP2 to terminate the FM (Lenhard et al., 2001; Zhao et al., 2007). AG promotes the binding of ARF3 to the *WUS* locus *in vivo* and *ARF3* is a direct target of AP2 (Liu et al., 2014).

At stages 5–6 of floral development, ARF3 mediates AG to promote FM determinacy by repressing cytokinin biosynthesis and signaling (Zhang et al., 2018b; Lee et al., 2019; Xu et al., 2019) (**Figure 2C**). Exogenous cytokinin treatment enhances the FM determinacy defect of *ag-10* mutants by modulating the temporal termination of *WUS* expression and cell division. ARF3 directly represses the expression of *IPT3*, *5*, and *7*, cytokinin biosynthetic genes, and indirectly represses *LONELY GUYs* (*LOGs*) to fine-tune cytokinin levels and activity (Cheng et al., 2013; Zhang et al., 2018b; **Figure 2G**). Furthermore, ARF3 directly inhibits the expression of cytokinin receptor genes like *AHK4* to regulate the perception of cytokinin in OC regions of the FM (Schaller et al., 2015; Zhang et al., 2018b; **Figure 2G**). Additionally, ARF3 regulates FM determinacy in a manner that depends on its ability to move between cells. The non-mobile ARF3 (ARF3-nls-GFP) only partially rescues the *ag-10 arf3-29* double mutant phenotype (Zhang et al., 2022), but *ARF3:ARF3-GFP ag-10 arf3-29* displayed full complementation (Liu et al., 2014; Zhang et al., 2018b). However, the function of ARF3 in FM determinacy is irrelevant to its function in organ patterning. Considering that *ag-10 kanadi1* (*kan1*) *kan2* triple mutant flowers have no FM determinacy defects, the KANs-ARF3 complex is not responsible for FM determinacy (Zheng et al., 2018).

## 5 ARF3’s role in pattern formation

**Figure 3:**
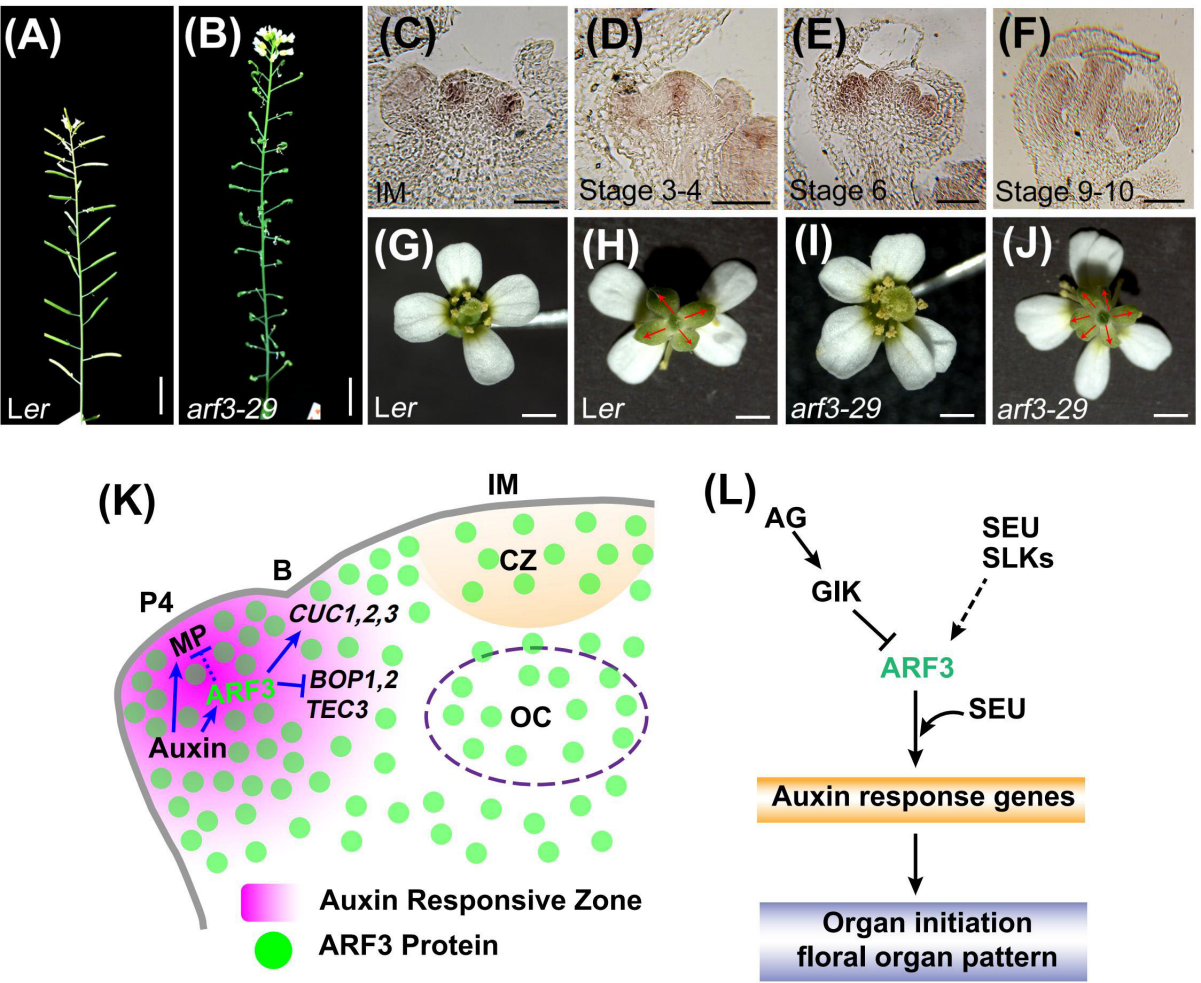
ARF3 controls phyllotaxy and floral organ patterning cell autonomously. **(A, B)**, Representative images of inflorescence stems for the indicated genotypes (from Zhang et al., 2022, provided by Dr. Ke Zhang). Scale bars = 1 cm. **(C–F)**, *ARF3* expression in IM **(C)** and FM at stage 3 **(D)**, stage 6 **(E)** and stage 9–10 **(F)** examined by *in situ hybridization* (from Zhang et al., 2022, provided by Dr. Ke Zhang). Bars = 50 μm. **(G–J)**, The number of floral organs in the indicated genotypes. Compared with Ler **(G, H)**, *arf3* flowers have more sepals, petals and stamens **(I, J)**. The sepals are marked by red arrows. Bars = 1 mm. **(K)**, **(A)** model of phyllotactic patterning control by ARF3. Auxin promotes *ARF3* in floral primordia, where it regulates meristem–organ boundary-specific genes (*CUC1–3*, *BOP1–2*, and *TEC3*) and *MP* in a cell-autonomous manner. Figures adapted from (Zhang et al., 2022). P4, primordia of flower buds at different developmental stages; **(B)**, meristem–organ boundary. Blue solid arrows indicate direct and indirect positive regulation. Blue solid and dashed block lines indicate direct and indirect negative regulation, respectively. **(L)**, A model of floral organ patterning control by ARF3. ARF3 cooperates with SEU to control organ initiation and floral organ pattern by regulating auxin response genes, and *ARF3* is inhibited by GIK directly and promoted by SEU and SLKs. Solid and dashed arrow indicates positive regulation. Block lines indicate negative regulation.

The regularity of plant architecture and morphology is a striking and mysterious phenomenon. The lateral organs, such as leaves, flowers, and floral organs, are arranged in regular patterns around a central axis (Reinhardt, 2005; Traas, 2013; Bartlett and Thompson, 2014). Leaves and flowers arise from the meristem in stereotypical patterns, known as phyllotaxis, which are related to the divergence angle between successive organs, most commonly approximating 137.5° (Smith et al., 2006; Okabe, 2012). The floral organs also exhibit a characteristically symmetrical arrangement, with their number, position, and shape being precisely regulated (Reinhardt, 2005; Thomson and Wellmer, 2019). *arf3* mutants exhibit obvious abnormalities in phyllotactic patterning, floral organ patterning, floral organ number, the patterning of abaxial tissues, and the establishment of apical and basal boundaries in primordia (**Figures 3A, B, G–J**; see section 4 for details).

### 5.1 Formation of phyllotactic patterning

Although many mathematical, physical, and chemical models have been proposed to explain the mechanism of phyllotactic pattern formation, the regular initiation of organs and their spatial and temporal positioning is the basis for the formation of phyllotactic patterns from a developmental biology perspective (Reinhardt, 2005; Smith et al., 2006; Traas, 2013; Bergeron and Reutenauer, 2019). The phytohormone auxin plays a critical role in establishing phyllotactic patterns by promoting organ initiation (Prasad et al., 2011; Bhatia et al., 2016). Loss-of-function mutations in boundary genes such as *CUP-SHAPED COTYLEDON2* (*CUC2*), *REPLUMLESS* (*RPL*), and *BELLRINGER* (*BLR*), which are involved in the formation of meristem-to-organ boundaries, often cause altered phyllotactic patterning (Byrne et al., 2003; Peaucelle et al., 2007; Bencivenga et al., 2016). *arf3* mutant plants display dramatically altered phyllotaxis. ETT^C2S^, an auxin-insensitive *ett* line, has a phyllotaxy defect, suggesting that ARF3 functions in an auxin-dependent manner (Simonini et al., 2017). Recently, evidence has emerged that ARF3 shares a common target gene, *TARGETS UNDER ETTIN CONTROL3* (*TEC3*), with RPL that regulates phyllotactic patterning (Simonini et al., 2017). Furthermore, ARF3 can directly regulate the expression of boundary-specific genes, including *CUC3*, *BLADE ON PETIOLE* (*BOP1*), and *BOP2*, to control phyllotaxy in a cell-autonomous manner (**Figure 3K**). Both ARF3-GFP and ARF3-nls-GFP can occupy the *CUC3*, *BOP1*, *BOP2*, and *TEC3* loci with similar enrichment levels in the *arf3* background. In *arf3-29*, *CUC1*, *CUC2*, and *CUC3* expression were lower than in the corresponding wild-type plants, while the transcript levels of *BOP1*, BOP2, and TEC3 were higher (Zhang et al., 2022). Therefore, ARF3 regulates boundary specific genes to determine the location of primordium initiation and thus influence phyllotactic patterning.

### 5.2 Formation of floral organ pattern

During flower development, FM and organ identity genes *LEAFY* (*LFY*) and *APETALA1* (*AP1*)/*CAULIFLOWER* (*CAL*) play key roles in the patterning of FMs by setting the spatial limits of expression of floral organ identity genes, such as *AP1*, *AP3*, *PISTILLATA* (*PI*), *AG*, and *SEPALLATA* (*SEP*) (John L. Bowman et al., 1989; Lee et al., 1997; Lenhard et al., 2001; Lohmann et al., 2001; Krizek and Fletcher, 2005; Chae et al., 2008; Alvarez-Buylla et al., 2010;). An “ABC” model, later extended to an “ABCDE” model, was formulated that proposes floral organ identity genes function combinatorially (Coen and Meyerowitz, 1991; Krizek and Fletcher, 2005; Thomson and Wellmer, 2019).

*ARF3* displays a complex expression pattern during early-stage floral development (**Figures 3C–F**). For example, *ARF3* mRNA is detected throughout the FM during stages 1-2. In stages 3-4, it is expressed in incipient stamen and gynoecium primordia. In stage 5-7, it concentrated in the abaxial side of petal, stamen, and gynoecium primordia (Sessions et al., 1997; Zhang et al., 2022). In line with its expression patterns, *ARF3* regulates the FM and floral organ initiation, and loss of function in *ARF3* leads to abnormal floral patterning. For example, the sepals and petals numbers are increased, while the number of stamens decreases (Sessions et al., 1997; Zhang et al., 2022). *SEUSS* (*SEU*) encodes a protein that interacts with ARF3 to affect auxin responses and facilitate floral organ patterning and growth (Pfluger and Zambryski, 2004; **Figure 3L**). In addition, three *SEU*-*LIKE* genes (SLK1, 2, and 3) have a degree of functional redundancy with SEU, and the expression level of *ARF3* was reduced in *seu slk1* inflorescences (Bao et al., 2010; **Figure 3L**).

## 6 ARF3’s role in gynoecium morphogenesis

ARF3 plays a prominent role in gynoecium patterning and identity (Simonini and Ostergaard, 2019). Wild-type Arabidopsis gynoecia are composed of a stigma-and-style-capped bilocular ovary on an unelongated internode. The ovary has two medial furrows and two lateral valves, and is the largest region of the gynoecium (Sessions et al., 1997; Roeder and Yanofsky, 2006). In *arf3* (*ett*) mutants, the valve tissue disappears basally, being replaced by structures intermediate between the abaxial style and internode. These aberrant phenotypes indicate ARF3 participates in apical/basal axis patterning of the gynoecium. (Sessions and Zambryski, 1995; Sessions et al., 1997). However, abaxial/adaxial polarity is also affected in *ett* gynoecia where an everted transmitting tract tissue develops in outgrowths on the exterior of the gynoecium (Nemhauser et al., 2000; Roeder and Yanofsky, 2006).

### 6.1 Formation of the apical/basal axis of gynoecia

**Figure 4:**
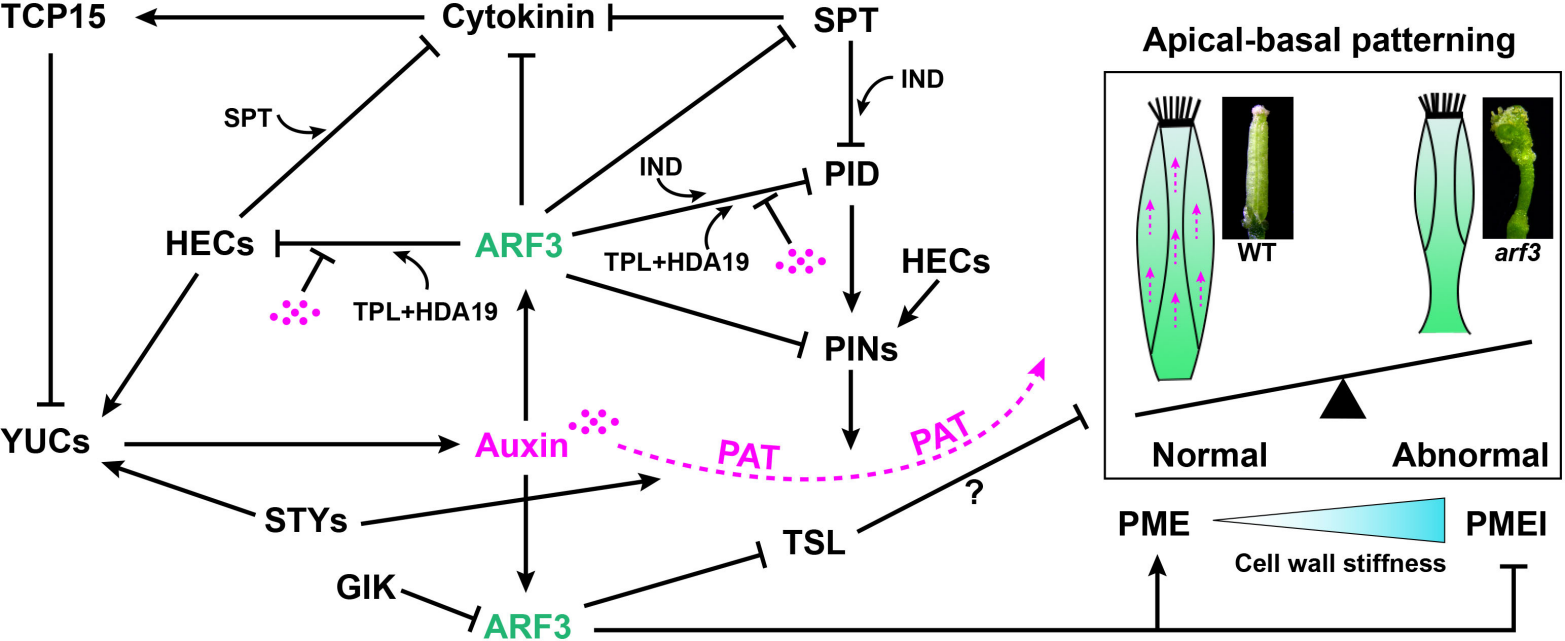
A model of the ARF3-mediated regulatory network in the apical/basal axis formation of gynoecia. Solid arrow indicates positive regulation. Block lines indicate negative regulation. Dashed arrow indicates polar auxin transport (PAT). The magenta dots indicate auxin.

*ARF3* mRNA is expressed in a ring around the FM preceding the formation of gynoecia at stage 5. The upper and bottom edges of the ring are required for proper specification of the apical and basal boundaries, respectively (Roeder and Yanofsky, 2006). Previous studies have shown auxin gradients play a key role in establishing the apical/basal axis of a gynoecium. (Nemhauser et al., 2000; Hawkins and Liu, 2014; Sehra and Franks, 2015; Marsch-Martinez and de Folter, 2016). Treatment with N-1-naphthylphthalamic acid (NPA), an inhibitor of polar auxin transport, promotes *ett*-like phenotypes. ARF3 affects the auxin gradient by negatively regulating *PIN-FORMED1* (*PIN1*) and *PIN3* auxin efflux transporters (Simonini et al., 2017), and two basic helix-loop-helix (bHLH) transcription factors, *SPATULA* (*SPT*) and (*HEC1*) (Heisler et al., 2001; Moubayidin and Ostergaard, 2014; Schuster et al., 2015; **Figure 4**). HEC1 is able to interact with SPT in yeast, and they act together to buffer auxin and cytokinin signals during gynoecium development (Schuster et al., 2014; Schuster et al., 2015; **Figure 4**). HEC1 can stimulate auxin biosynthesis and directly promotes the expression of *PIN1*and *PIN3* auxin efflux transporters. It also inhibits CK signals by promoting the expression of type-A ARRs. TEOSINTE BRANCHED1-CYCLOIDEA-PCF 15 (TCP15), a class I TCP transcription factor, can also modulate cytokinin and auxin responses during gynoecium development. TCP15 can not only affect auxin homeostasis by inhibiting auxin synthesis-related *YUC* genes, but it can also be induced by cytokinin and modulates the expression of cytokinin-responsive genes (Steiner et al., 2012; Lucero et al., 2015; **Figure 4**). The SPT- IND complex, however, directly represses the expression of *PID*, which modulates PIN polarization (Girin et al., 2011; Schuster et al., 2015). Recently, Sara Simonini et al., showed ARF3 and IND interact to form a complex that mediates gynoecium patterning *via* PID transcriptional control, and auxin affects the activity of this complex (Simonini et al., 2016). Furthermore, Andre´ Kuhn et al., showed under low auxin conditions an ARF3-TPL-HDA19 complex binds to the promoter of *PID* and *HEC1* keeping their chromatin environments repressed, through de-acetylation, while high nuclear auxin concentrations abolish the ETT-TPL-HDA19 complex through direct ARF3-auxin interaction (Kuhn et al., 2020). TOUSLED (TSL), a nuclear serine/threonine protein kinase, plays a role in apical tissue formation (Roe et al., 1993; Roe et al., 1997). In *ett* mutants, *TSL* is ectopically expressed in the stylar medial tissue and valves, which indicates ARF3 limits *TSL* expression to the apex of gynoecia to maintain apical boundary identity (Roe et al., 1997; Sehra and Franks, 2015). STYLISH1 (STY1) and STY2 are members of the SHI/STY-family, and *sty1-1 sty2-1* double mutants show a reduced production of stylar xylem and a basalized point of medial vein bifurcation in the gynoecium (Kuusk et al., 2002). STY1 synergistically interacts with ARF3 during apical-basal patterning and apical fusion of gynoecia by controlling YUCCA-mediated auxin biosynthesis and polar auxin transport (PAT) (Sohlberg et al., 2006; **Figure 4**). *GIK*, as a direct target of AG, negatively regulates *ARF3* expression and plants overexpressing *GIK* displays stigmatic tissue outgrowth, short valves, and bipartite stigmas with ectopic ovules, closely resembling *ett* mutants (Ng et al., 2009; **Figure 4**).

Recently, evidence has shown ARF3 modulates gynoecium morphogenesis *via* cell wall dynamics (Andres-Robin et al., 2018). Amélie Andres-Robin et al., proposed ARF3 functions by reducing cell wall stiffness through the covalent modification of pectin. ARF3 acts to increase pectin methylesterase (PME) activity by stimulating PME genes and repressing pectin methylesterase inhibitor (PMEI) gene expression in the valves (Pelloux et al., 2007; Andres-Robin et al., 2018; Wormit and Usadel, 2018). It is possible that ARF3 affects the activity of PME/PMEI indirectly, and it is in fact a consequence of other processes affected by ARF3 such as auxin dynamics (Nemhauser et al., 2000; Andres-Robin et al., 2018; **Figure 4**).

### 6.2 Formation of the abaxial/adaxial axis of the gynoecium

In leaf and gynoecium primordia, *ARF3* transcription is restricted to the abaxial cells during stages 5-8 and this is essential in determining adaxial-abaxial polarity. (Sessions et al., 1997; Iwasaki et al., 2013; Liu et al., 2014; Husbands et al., 2015). Previous reports revealed transcription of *ARF3* and its homolog *ARF4* is restricted to the abaxial side of the leaf by TAS3-derived trans-acting small interfering RNAs (tasiRNAs) known as tasiR-ARFs (Williams et al., 2005; Hunter et al., 2006a). The tasiR-ARFs are distributed on the adaxial side, forming a gradient towards the abaxial side that limits *ARF3* and *ARF4* to the abaxial side (Garcia et al., 2006; Chitwood et al., 2009). Recently, *ARF3:ARF3m-GFP*, a TAS3 tasiRNA-insensitive transgene, led to a ubiquitous distribution of *ARF3* mRNA in gynoecium primordia and ovules, suggesting the expression pattern of *ARF3* is regulated by tasiR-ARFs during gynoecium development (Liu et al., 2014; Su et al., 2017).

**Figure 5:**
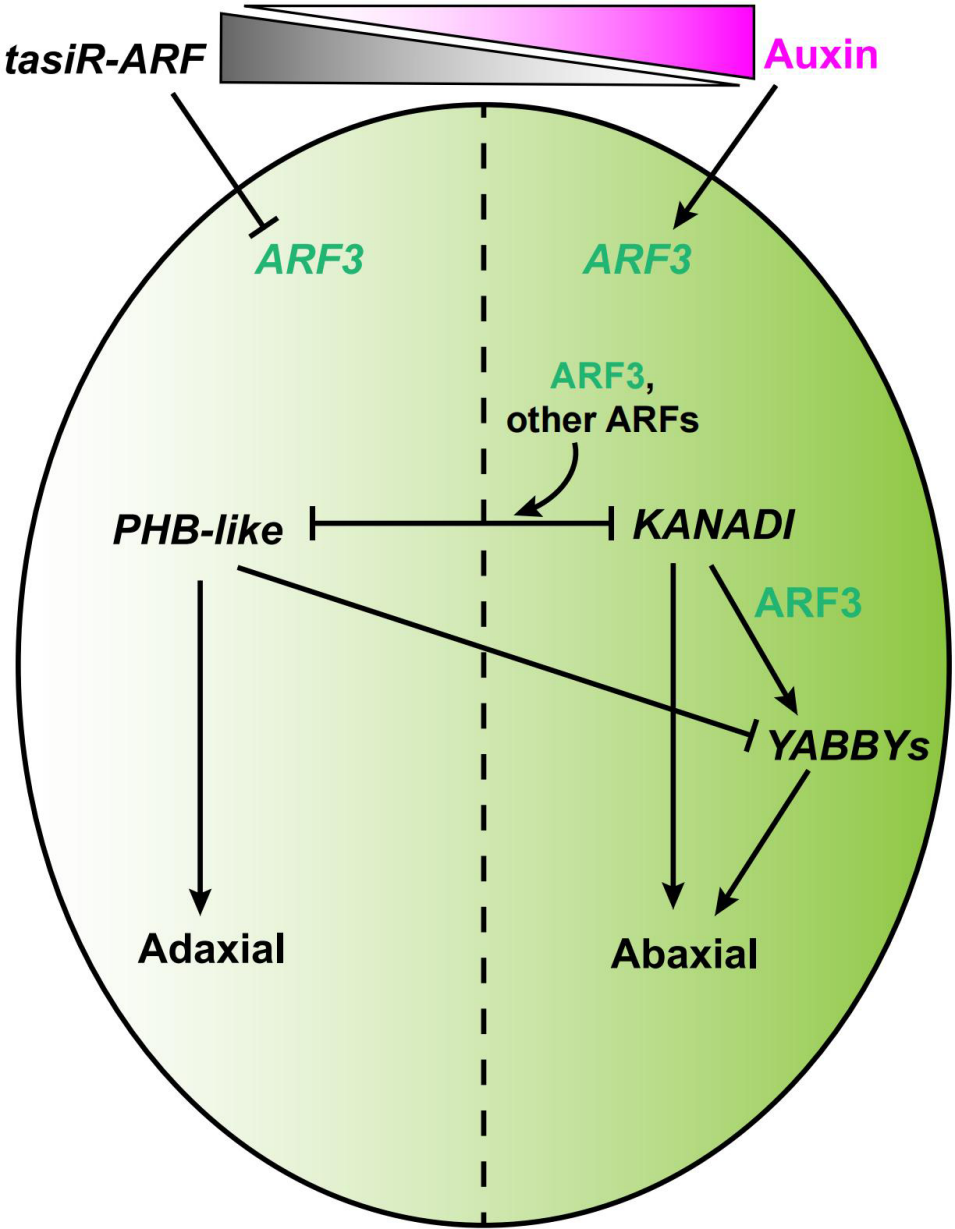
A model of the ARF3-mediated regulatory network in the abaxial/adaxial axis formation of gynoecia. ARF3 is indicated in green, auxin in magenta, and tasiR-ARF3 in gray. Solid arrow indicates positive regulation. Block lines indicate negative regulation.

ARF3, cooperating with ARF4, regulates the adaxial/abaxial polarity of valves in conjunction with KANADI (KAN) (Pekker et al., 2005; **Figure 5**). The GARP transcription factor KAN1 specifies abaxial polarity in both the gynoecium and the leaves (Eshed et al., 2001; Kerstetter et al., 2001). ARF3 interacts physically with KAN1 in yeast, and the activity of KAN1 is required for ARF3 (Pekker et al., 2005; Kelley et al., 2012). *YABBY* genes are expressed on the abaxial side of all lateral organ primordia and are capable of inducing the differentiation of abaxial cell types when expressed adaxially (Eshed et al., 1999; Sawa et al., 1999; Siegfried et al., 1999). FILAMENTOUS FLOWER (FIL), a member of the YABBY family, is a target of ARF3, and both KAN and ARF3 promote the expression of *FIL* (Eshed et al., 2001; Garcia et al., 2006; **Figure 5**).

## 7 ARF3’s role in ovule development and self-incompatibility

Carpel fusion creates a protected cavity that houses the ovules and the placenta. Ovules develop from a ridge of tissue that originates on the adaxial side of the gynoecium at stages 8-10 (Bowman et al., 1999). The ovule primordia arise as finger-like projections from the placenta, and the megaspore mother cell (MeMC) is derived from a sub-epidermal cell at the distal end of the ovule primordium, which is committed to generating the haploid, multicellular female gametophyte (Drews and Koltunow, 2011). Recent studies have shown ARF3 is involved in ovule development and mediates the inhibition of *TAS3* during MeMC formation (Su et al., 2017; Pinto et al., 2019). The THO/TREX complex plays a conserved role in processing and transporting long RNA molecules during small interfering RNA (siRNA) biosynthesis (Chavez et al., 2000; Strasser et al., 2002; Yelina et al., 2010). Mutations in THO complex members, such as *TEX*, *HPR1*, and *THO6*, can inhibit megaspore mother cell fate by accelerating the biogenesis of tasiRNAs that repress *ARF3* expression. Loss of function in *TEX1*, *RDR6*, and *TAS3* leads to supernumerary MeMC formation and the ectopic expression of *ARF3* can phenocopy these MeMC defects (Su et al., 2017; Lora et al., 2019).

**Figure 6:**
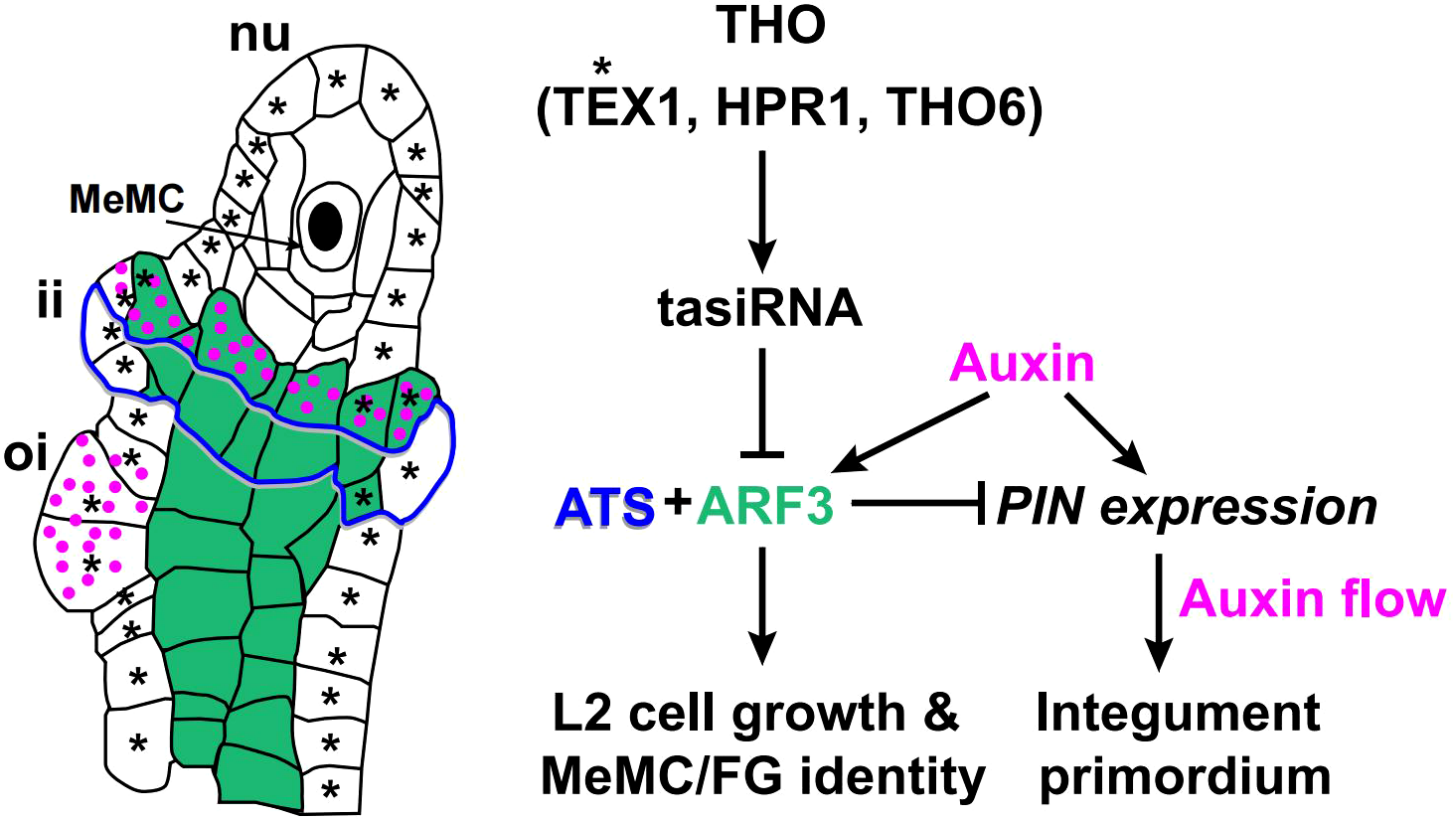
A model of the ARF3-mediated regulatory network during ovule development. ARF3 is indicated in green, auxin in magenta, tasiR-ARF in gray, and ATS in blue with shades of gray (Kelley et al., 2012). Green area indicates the distribution of ARF3 (Su et al., 2017; Lora et al., 2019). The magenta dots indicate the distribution of auxin. Blue lines with shades of gray indicate the distribution region of ATS. Asterisks indicate the distribution of TEX1. Solid arrows indicate positive regulation. Block lines indicate negative regulation. nu, nucellus; ii, inner integument; oi, outer integument.

ARF3 also directly interacts with KAN4/ABERRANT TESTA SHAPE (ATS) to define the boundary between integument primordia (Kelley et al., 2012; Shirley et al., 2019; **Figure 6**). The expression pattern of *ARF3* overlaps with *ATS*, being restricted to the inner integument during ovule development (Kelley et al., 2012; Lora et al., 2015). The *ats* mutant forms aberrant ovules, in which the inner and outer integument cell layers grow as a single fused structure (McAbee et al., 2006; Kelley et al., 2012). The ovule phenotype of *ett* resembles *ats*, and the ovules of *ats ett* double mutant plants showed no phenotypic differences to either single mutant. Thus, loss of either ATS or ETT is sufficient to disrupt a common regulatory pathway that is mediated by both TFs. (Kelley et al., 2012). Dior R. Kelley et al. proposed the ATS-ARF3 module might be directly linked to auxin signaling by restricting PIN activity and, thus, auxin flow (Benkova et al., 2003; Bencivenga et al., 2012; Kelley et al., 2012; **Figure 6**).

Auxin gradients also modulate ovule and integument development by controlling the activity of ARF proteins, such as MONOPTEROS (MP)/ARF5 and ARF3 (Galbiati et al., 2013; Sehra and Franks, 2015; Marsch-Martinez and de Folter, 2016; Simonini et al., 2016). ARF3 regulates ovule integument development in an auxin-sensitive fashion. Previous research has shown *ETT : ETT-GFP* can complement the integument defect of *ett*, but *ETT : ETT^2C-S^*, an auxin-insensitive variant, was not able to rescue this defect (Simonini et al., 2016).

Self-incompatibility is one of the most common mechanisms by which angiosperms prevent self-fertilization and enforce outcrossing. A genetic self-incompatibility (SI) allows cells in the pistil to recognize and specifically prevent “self” pollen from affecting fertilization (Nasrallah, 2019). However, Arabidopsis is highly self-fertile due to it lacking functional alleles of two genes, the S-locus receptor kinase (SRK) and the S-locus cysteine-rich protein (SCR), which function in SI in the Brassicaceae (Tantikanjana and Nasrallah, 2012; Nasrallah, 2019). Tantikanjana and Nasrallah demonstrated that ARF3 acts non-cell-autonomously as a regulator of SI by mediating TAS3 tasiRNA to enhance SI. *ARF3:ARF3mut*, however, a tasiRNA-insensitive variant, enhances SI whereas loss-of-function *ett* mutations abolish SI (Tantikanjana and Nasrallah, 2012).

## 8 Conclusions and future prospects

ARF3 is a noncanonical auxin response factor with a special structure, is regulated at multiple levels, and performs a wide range of functions. Because ARF3 lacks the C-terminal domain (PB1 domain), its response to auxin does not depend on the AUX/IAA pathway. In addition to inducing the transcription of *ARF3*, auxin affects its regulation of target genes. Studies suggest that ARF3 binds auxin and regulates its target genes in either an auxin-dependent or -independent manner. Aside from transcriptional regulation, *ARF3* is also regulated by tasiR-ARF, translation reinitiation, and DNA methylation. Recent reports have shown that ARF3 can also modulate SAM maintenance through intercellular migration. Sophisticated regulation often implies complex functionality. ARF3 functions in both the regulation of patterning formation and meristem activity. ARF3 can affect phyllotactic patterning by changing the divergence angle between successive floral primordia, can control floral organ patterning by changing the number and arrangement of floral organs, and plays an important role in the regulation of apical/basal and abaxial/adaxial organ polarity. ARF3-mediated auxin signaling contributes to the fine-tuning of meristem activity, and is involved in SAM homeostasis and FM determinacy. Future research into the biological function and regulatory mechanisms of ARF3 may (1) further clarify and verify the auxin-response mechanism of *ARF3*, for example, by uncovering the mechanism by which auxin regulates *ARF3* transcription, and the mechanism by which auxin affects the interaction between ARF3 and cooperators to modulate target gene expression; (2) explore the molecular mechanism that regulates ARF3 intercellular migration, screening for the domains that affect the spatial patterning of ARF3 protein; and (3) analyze the molecular mechanisms by which ARF3 regulates meristem homeostasis to better understand this regulatory network.

## Author contributions

KZ and XL conceived and designed the project. YF and HZ wrote the paper. YM drew the model. CL provided some suggestions for the paper. All authors contributed to the article and approved the submitted version.
